# Human Usutu Virus Infection with Atypical Neurologic Presentation, Montpellier, France, 2016

**DOI:** 10.3201/eid2405.171122

**Published:** 2018-05

**Authors:** Yannick Simonin, Olivier Sillam, Marie J. Carles, Serafin Gutierrez, Patricia Gil, Orianne Constant, Marie F. Martin, Gilda Girard, Philippe Van de Perre, Sara Salinas, Isabelle Leparc-Goffart, Vincent Foulongne

**Affiliations:** Université de Montpellier, Montpellier, France (Y. Simonin, O. Constant, M.F. Martin, P. Van de Perre, S. Salinas, V. Foulongne);; Université de Montpellier Hôpital, Montpellier (O. Sillam);; Nimes University Hospital, Nimes, France (M.J. Carles);; Centre de Coopération Internationale en Recherche Agronomique pour le Développement, Montpellier (S. Gutierrez, P. Gil);; Institut de Recherche Biomédicale des Armées, Marseille, France (G. Girard, I. Leparc-Goffart)

**Keywords:** Usutu virus, USUV, viruses, flavivirus, human infections, atypical neurologic presentation, meningitis/encephalitis, Bell’s palsy, idiopathic facial paralysis, Montpellier, France

## Abstract

Infection with Usutu virus (USUV) has been recently associated with neurologic disorders, such as encephalitis or meningoencephalitis, in humans. These findings indicate that USUV is a potential health threat. We report an acute human infection with USUV in France putatively associated with a clinical diagnosis of idiopathic facial paralysis.

Usutu virus (USUV) is a mosquitoborne flavivirus. This virus was detected in South Africa in 1959 and is maintained through an enzootic cycle involving birds as the main amplifying reservoir hosts and ornithophilic mosquito species as vectors (mainly *Culex* spp.) (*1*). Mammals, including humans, are incidental dead-end hosts. Although USUV was considered a tropical or subtropical virus, it was recently introduced in central and western Europe. Emergence of USUV in Europe was reported in Austria in 2001, but retrospective analyses have suggested an earlier introduction because several epizootics and small outbreaks among local birds have been suspected since 1996 (*1*). In 2016, a large USUV epizootic was reported in Belgium, France, Germany, and the Netherlands (*2*).

The zoonotic potential of USUV was initially described in the Central African Republic and recently confirmed in Europe by reports of neuroinvasive infections caused by this virus (*1*). Further evidence of probable human infections was demonstrated by seroprevalence studies on healthy blood donor samples: prevalence of 1.1% in Italy (*3*) and 0.02% in Germany (*4*). Furthermore, a recent blood donor screening in Germany identified an acute USUV infection (*5*). Although human infections have not been identified in France, deaths of birds during 2015–2016 confirmed USUV circulation (*6**,**7*).

Moreover, recent data have shown a high prevalence (7%) of USUV in *Culex pipiens* mosquitoes in the Rhone River delta, a region also called Camargue, in 2015 (M. Eiden et al., unpub. data). Camargue is a landscape of wetlands that hosts a diversity of wild bird species, including migratory birds, and diverse mosquito populations. This environment could potentially favor transmission of USUV to humans, similar to that for West Nile virus (WMV) in this area (*8*).

We investigated the zoonotic potential of USUVs and WNVs in France by a retrospective flavivirus molecular survey of cerebrospinal fluid (CSF) samples collected in 2016 during the period of maximum mosquito activity (May–November). Samples were obtained from patients with infectious or neurologic syndromes in 2 towns near Camargue. One CSF sample was positive for USUV RNA. We report detection of an acute human infection with USUV in France associated with an unexpected clinical diagnosis of idiopathic facial paralysis.

## The Study

We retrospectively screened a collection of RNA extracts stored at −80°C by using a modified consensual panflaviviruses assay (*9*) with a One-Step RT-PCR Sybr-Green Mixture (QIAGEN, Hilden, Germany). Extracts were obtained from 666 CSF samples collected at the Université de Montpellier Hôpital (Montpellier, France) and Nîmes University Hospital (Nîmes, France) during May–November 2016. Samples are part of a registered systematic collection established for epidemiologic purposes during the surveillance period for risk of infection with arboviruses (May–November) in a region of southern France that has *Aedes albopictus* mosquito vectors (Table). RNA extracts from samples with a previous probable bacterial or viral etiology were not assessed.

**Table:** Clinical conditions or symptoms associated with microbial investigations of cerebrospinal fluid samples for infections with arboviruses, France*

Condition	No. (%) positive samples
Meningitis/encephalitis	277 (41.6)
Neurologic disorders†	233 (34.9)
Febrile syndrome	108 (16.2)
Other	48 (7.0)
Total	666 (100.0)

*Samples collected at the Université de Montpellier Hôpital (Montpellier, France) and Nîmes University Hospital (Nimes, France) during May–November 2016. †Including (if >2%) convulsion/epileptic seizure (25%); paralysis/paresthesia/polyradiculoneuritis/motor loss/palsy (31%); myelitis (2%); vascularitis (3%); encephalopathy (6%); Guillain-Barré syndrome (4%), headache (15%); and confusion (10%).

**Figure 1:**
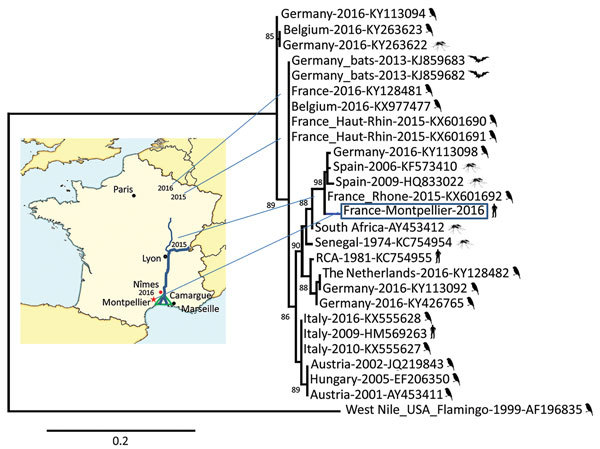
Phylogenetic relationship of the France-Montpellier-2016 strain of Usutu virus (USUV) (box; GenBank accession no. LT854220), isolated from a 39-year-old man in Montpellier, France, who had an atypical neurologic presentation, compared with other USUV strains based on the partial nonstructural protein 5 gene sequence. USUV sequences are shown with their country of isolation, year of isolation, and GenBank accession numbers. Hosts from which the strains were detected (bird, mosquito, bat, or human) are shown next to strain names. Analysis was processed through the French phylogeny website (http://www.phylogeny.fr). Nucleotide sequences were aligned by using MUSCLE software (https://www.ebi.ac.uk/Tools/msa/muscle/). The phylogenetic tree was constructed by using the maximum-likelihood method in PhyML (http://www.atgc-montpellier.fr/phyml/). One hundred bootstrap datasets with random sequence addition were computed to generate a consensus tree drawn with TreeDyn software (http://www.treedyn.org/) and rooted with a West Nile virus sequence (GenBank accession no. AF196835). Numbers along branches are bootstrap values. Map shows locations where USUV strains were detected in France during 2015–2016. Scale bar indicates nucleotide substitutions per site.

One sample showed a positive reverse transcription PCR (RT-PCR) result for panflaviruses (cycle threshold 33). Subsequent Sanger dideoxy sequencing with amplification primers for a 260-bp nonstructural protein 5 gene sequence identified a USUV RNA sequence. Phylogenetic analysis based on this partial sequence, previously shown to accurately discriminate USUV lineages (*2*,*10*), identified a strain closely related to viruses that were circulating in birds in southern Europe (Figure 1).

**Figure 2:**
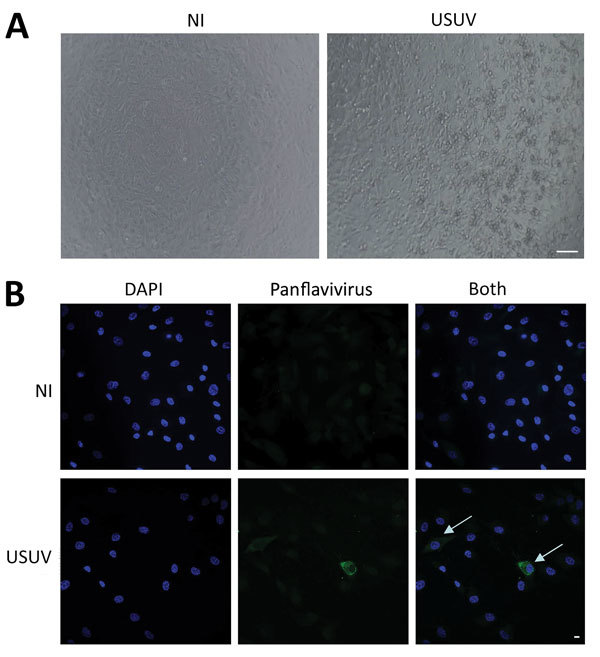
Cerebrospinal fluid sample of a 39-year-old man in Montpellier, France, infected with USUV who had an atypical neurologic presentation. The sample was amplified for 6 days on C6/36 cells, and the supernatant was used to infect Vero cells or primary human astrocytes. A) Cytopathic effect (presence of adherent dead cells and absence of heaps; all dead cells were scattered) was observed at day 5 postinfection of a Vero cell culture. Scale bar indicates 100 μm. B) Mock or infected primary human astrocytes were fixed at day 4 postinfection and labeled with pan-flavivirus antibody (MAB10216, clone D1–4G2) by indirect immunofluorescence (green). Strong labeling was observed in some cells (arrows). Nuclei are labeled with DAPI (4′,6-diamidino-2-phenylindole) (blue). NI, not infected; USUV, Usutu virus. Scale bar indicates 10 μm.

We tested a remaining stored aliquot of a CSF sample for USUV by using a specific USUV RT-PCR (cycle threshold 30) (*11*). We inoculated this aliquot onto Vero cells and primary human astrocytes (*12*) after a round of amplification in C6/36 cells. Infected Vero cells showed a typical cytopathic effect (Figure 2, panel A) (*13*). We also detected infected astrocytes by immunofluorescence, thus demonstrating the presence of infectious viral particles (Figure 2, panel B). However, we detected no viremia in a blood sample by specific USUV RT-PCR and no specific USUV antibodies. Although virus and antibody dynamics are unknown for acute USUV infection, it is likely that these samples, collected 3 days after onset of symptoms, were collected too early to detect antibodies, as observed for other flaviviruses (*14*).

The case-patient was a 39-year-old man admitted to the Department of Neurology, Université de Montpellier Hôpital on November 10, 2016, because of sudden peripheral facial palsy and eyelid ptosis after prodromal dysgeusia. We obtained informed consent from the case-patient to publish his results.

The patient did not report any previous infectious signs or history of recent travel. Symptoms onset occurred 3 days before admission, and at the time of admission, he experienced gradual paresthesias of both right limbs and reported transient right upper limb palsy for ≈1 h. No other objective alteration of cranial nerves was detected during neurologic examination. Sensitivity was normal with presence of regular osteo-tendinous reflexes. Blood cell count, levels of liver enzymes, and renal function results were within reference ranges. No inflammatory or infectious syndromes were observed.

C-reactive protein level in a blood sample was 0.6 mg/L (reference value <5 mg/L). CSF protein level was 67 mg/dL (reference range 15–35 mg/dL). There was no pleiocytosis. Results of CSF cultures and routine PCRs were negative for herpes simplex virus, varicella zoster virus, and enteroviruses. Magnetic resonance imaging of the brain showed unremarkable results: no ischemic disorders on diffusion-weighted imaging and no enhancement with gadolinium in brainstem and cranial nerves. Results of nerve conduction studies on the 4 limbs were normal. The patient was given a diagnosis of idiopathic facial paralysis.

The patient was given corticoids, valaciclovir, and eye drops for prevention of keratitis. He was discharged 3 days later, and symptoms of facial palsy disappeared within a few weeks.

## Conclusions

Since the first major epizootic event in 2002, continuous geographic expansion of the range of USUV in Europe has been shown by reports of epizootics or small outbreaks from various countries in western Europe, with widespread activity of multiple lineages (*2*). Thus, USUV has likely become a potential human health concern and an increasing number of human infections have been described. Our report suggests an acute human USUV infection and shows that circulation of USUV involves a wider geographic distribution in France than reported (*2*). Phylogenetic analysis of the virus sequence isolated from the patient showed a strain probably related to the USUV/Spain strain, which has been detected in common blackbirds in France (*6*). Because our study region was near Camargue, detection of similar strains upstream and at the mouth of the Rhone River is consistent.

The zoonotic potential of USUV infection in Europe has been reported in a limited number of cases, including reports of encephalitis or asymptomatic cases (*1*). However, a retrospective study in a disease-endemic area in Italy showed that human USUV infection is not a sporadic event and showed a higher incidence than infection with WNV (*1*). For our case-patient, absence of evidence of an infectious syndrome associated with a clinical neuromuscular presentation of acute unilateral facial paralysis is atypical. However, because idiopathic facial paralysis, also known as Bell’s palsy, could be caused by ischemic, immune, and infective mechanisms (*15*) and the well-described neurotropism of some flaviviruses, the etiologic role of USUV must not be ruled out.

Our report of an acute human USUV infection in France reinforces the need for integrated surveillance in animals, vectors, and humans. The atypical clinical presentation remains an intriguing point that deserves more investigations and suggests that human USUV infections might display various clinical patterns and could have been underestimated.
